# Analytical Model for Load–Slip Relationship of Perfobond Shear Connector Based on Push-Out Test

**DOI:** 10.3390/ma12010029

**Published:** 2018-12-21

**Authors:** Shuangjie Zheng, Chen Zhao, Yangqing Liu

**Affiliations:** 1 College of Civil Engineering, Huaqiao University, Xiamen 361021, China; 2 Shanghai Municipal Engineering Design Institute (Group) Co., Ltd., Shanghai 200092, China; zhaochen@smedi.com; 3 Department of Bridge Engineering, Tongji University, Shanghai 200092, China; 1432232@tongji.edu.cn

**Keywords:** composite structure, perfobond connector, push-out test, load–slip relationship, analytical model

## Abstract

In composite structures, the perfobond connector is an alternative type of shear connector which consists of a steel plate with a certain number of holes. The load–slip relationship is critical for structural analysis and numerical simulation of composite structures using perfobond connectors. In this study, push-out tests were conducted on 72 specimens to obtain the load–slip behaviors of perfobond connectors. Based on the test results, parametric study was performed to analyze the effects of changing the hole geometry, the concrete strength, the configuration of the rebar in hole, the dimensions of the perfobond rib, and the size of the concrete slab. Furthermore, the characteristics and physical meanings of the load–slip curves were analyzed, and the limits and conditions for theoretical expressions were derived. Finally, an analytical model was proposed to express the load–slip relationship of perfobond connectors covering a wide range of design variables.

## 1. Introduction

Steel and concrete composite structures are widely used in bridges due to their favorable performance and economic cost. Various types of innovative composite structures have been developed in bridge structures, such as composite girders with corrugated steel webs [1], hybrid girders [2], pile cap strengthening [3], and composite trusses [4]. To optimize the structural behavior of composite structures, it is critical to ensure the shear connection between steel and concrete members. The shear connection is typically achieved by using different types of shear connectors, such as headed studs [5], perfobond connectors [6,7], Y-type perfobond connectors [8], T, T-block and T-perfobond connectors [9], composite dowels [10], I-shaped connectors [11], channel connectors [12], hat-shaped connectors [13], and bolted connectors [14]. Among these shear connectors, headed studs are the most common type of shear connectors used in composite bridges. However, specific welding equipment is essential for installing headed studs. Another disadvantage is the fatigue problems of weld under cyclic loading [15].

**Figure 1:**
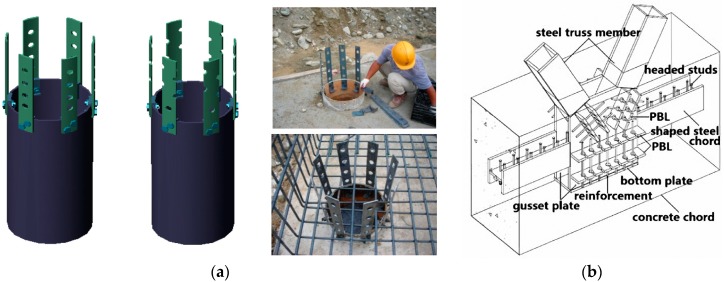
Engineering applications of perfobond connectors. (**a**) Pile cap strengthening [3]; (**b**) composite trusses [4].

To overcome the drawbacks of headed studs in installation and fatigue problems, an alternative shear connector named perfobond connector was first developed in a railway bridge project in the 1980s. The typical layout of a perfobond connector is a flat steel plate with a certain number of holes. Concrete dowels in the holes can resist shear and uplift forces between steel and concrete. In comparison with headed studs, perfobond connectors are much easier to install by fillet welding, and no obvious fatigue problems have been observed for perfobond connectors. Moreover, several researchers have reported that the perfobond connector has high shear stiffness and strength in push-out tests [16,17,18]. As a result, perfobond connectors are increasingly used in many types of composite bridge structures which need to carry great dynamic loads [3,4], as shown in Figure 1.

The structural behavior of composite structures is significantly influenced by the nonlinear behavior of shear connection, which leads to additional deformations in composite structures and causes load redistributions among the structural members [19,20]. As a result, the strength and the ductility of the composite structures may be decreased. To calculate the nonlinear behavior of shear connection, it is necessary to establish the load–slip relationship of shear connectors at the steel–concrete interface.

In previous researches, several expressions were proposed to predict the load–slip relationship of headed stud shear connectors [21,22]. These expressions were mostly derived from regression analysis of push-out test results. However, the load–slip response of perfobond connectors differs greatly from that of headed studs [5,6,7,8,9]. Due to variations in geometries and material properties, it is not suitable to duplicate these expressions for perfobond connectors. Japan Society of Civil Engineers (JSCE) suggested two expressions to derive the load–slip relationship of perfobond connectors without and with rebar in hole [23]. FIB recommended a general expression to fit the tested load–slip curves of shear connectors [24].

In this study, a total of 72 push-out tests were performed to obtain the load–slip behaviors of perfobond connectors. Based on the test results, parametric study was conducted to analyze the influences of hole geometry, the concrete strength, the configuration of the rebar in hole, the dimensions of the perfobond rib, and the size of the concrete slab. The characteristics and physical meanings of the load–slip curves were analyzed to derive the conditions for theoretical expressions. Finally, an analytical model was proposed to express the load–slip relationship of perfobond connectors.

## 2. Push-Out Test

### 2.1. Test Specimens

A total of 72 push-out tests were carried out to evaluate the shear behavior of the perfobond connectors. Table 1 presents the parameters of these push-out specimens, including the hole geometry (the hole diameter *d*, the hole length *d_l_*, and the hole height *d_h_*), the concrete strength *f_cu_*, the configuration of the rebar in hole (the existence of rebar, the diameter *d_s_*, and the yield strength *f_y_*), the dimensions of the perfobond rib (the rib thickness *t*, the rib height *h*, and the rib distance *e*), and the size of the concrete slab (the width *a* and the thickness *b*).

**Figure 2:**
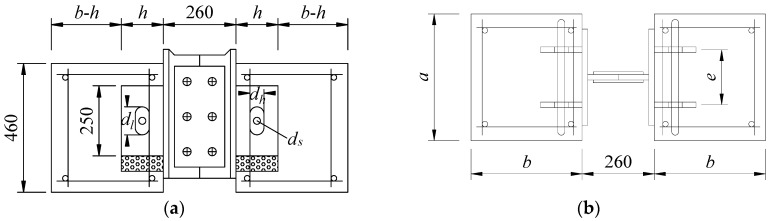

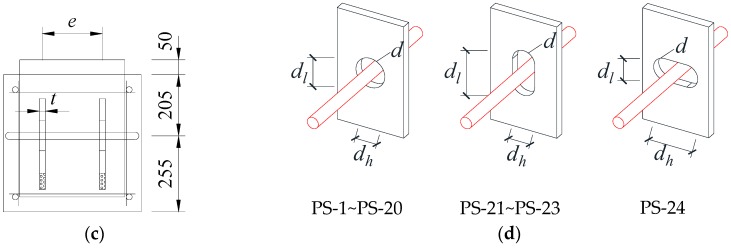
Layout of push-out test specimen. (**a**) Front view; (**b**) top view; (**c**) side view; (**d**) hole geometry.

These push-out specimens were fabricated referring to the standard push-out test in Eurocode 4 [25]. As shown in Figure 2, each specimen consisted of one steel H-beam, two concrete slabs, and four perfobond connectors with circular hole or long hole. A long hole was a combination of one rectangle and two semicircles. A Styrofoam was attached to the bottom of the perfobond ribs to eliminate the end-bearing resistance. The surface of the steel plate in contact with concrete was greased to prevent the bond. The general test results have been described by Zheng et al. [6,7].

### 2.2. Material Properties

As listed in Table 1, the concrete strength *f_cu_* was obtained from 150 mm concrete cube tests after a 28-day air curing period. The yield strength *f_y_* of the rebar in hole was determined by tension tests. The mean yield strength and tensile strength of the structural steel were 410.0 MPa and 545.0 MPa, respectively.

### 2.3. Test Setup and Instrumentation

**Figure 3:**
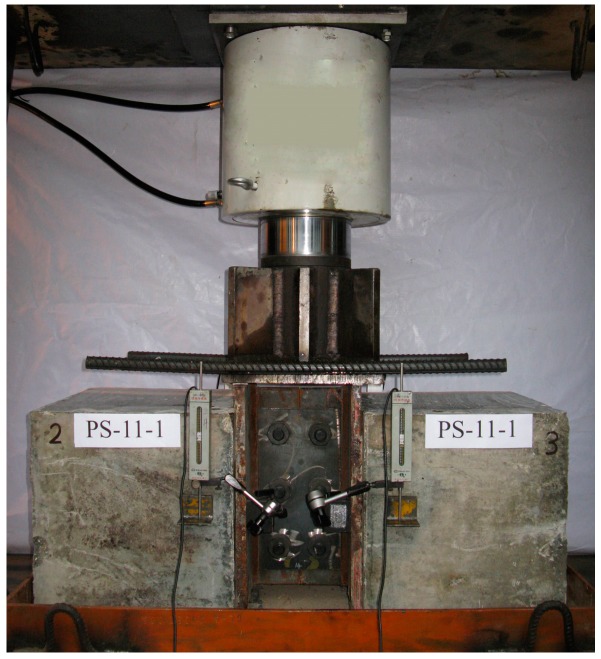
Test setup and instrumentation.

The push-out specimens were tested in a hydraulic loading machine with a 4000 kN capacity, as shown in Figure 3. In accordance with Eurocode 4 [25], the load was applied slowly in several steps to ensure that the specimen failure did not occur in less than 15 min. The slip between steel and concrete was measured by averaging the output of four gauges of 100 mm in length which were fixed at the level of the perfobond connectors. The load and the slip were both continuously recorded. As a result, the load–slip curves of the test specimens could be obtained.

## 3. Test Results

### 3.1. Failure Modes

**Figure 4:**
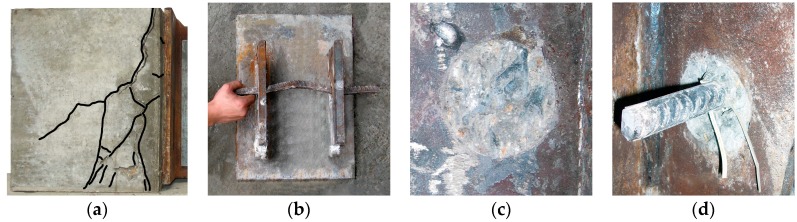
Failure modes. (**a**) Crack in concrete; (**b**) yield of rebar; (**c**) shear in dowel without rebar; (**d**) shear in dowel with rebar.

As shown in Figure 4, the failure modes of the perfobond connectors were mainly characterized by failure in the concrete. The crack initially appeared near the shear connectors and gradually spread out across the concrete slab. After testing, the concrete slabs were demolished to investigate the behavior of the perfobond connectors. With or without rebar in holes, the concrete dowels failed in shear. The rebar in hole yielded due to large bending and shear deformation.

### 3.2. Shear Mechanism

**Figure 5:**
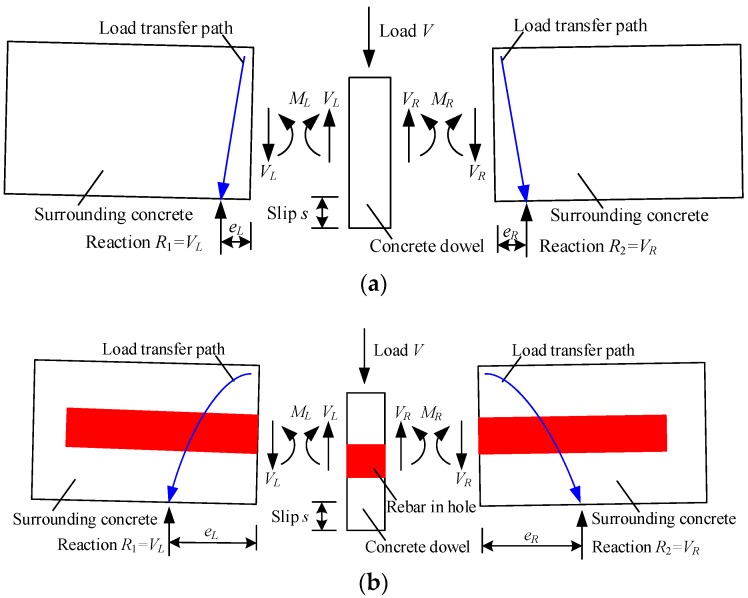
Shear mechanism. (**a**) Without rebar in hole; (**b**) with rebar in hole.

According to the failure modes observed in push-out tests, the shear mechanism of the perfobond connectors could be obtained and is depicted in Figure 5. The load transfer between the steel and concrete at the local area of the concrete dowel was depicted to compare the shear mechanism of perfobond shear connectors with and without rebar in hole. As the load *V* was applied to the perfobond connector, shear and bending deformation caused the slip *s* to increase between the concrete dowel and the surrounding concrete. Meanwhile, the shear forces *V_L_*, *V_R_* and the moments *M_L_*, *M_R_* were induced at the steel–concrete interface.

As shown in Figure 5a, when no rebar was provided in hole, the concrete dowel had small resistance to the moments *M_L_* and *M_R_*, which restricted the increase of the slip *s* at failure. The load transfer path was almost straight downward. A small region of the surrounding concrete was involved to provide the reactions *R*_1_ and *R*_2_. As a result, without rebar in hole, the shear strength and the slip ductility of the perfobond connectors were limited by the concrete strength.

When a rebar was provided in hole, as shown in Figure 5b, the resistance to the moments *M_L_* and *M_R_* was greatly increased. Larger shear and bending deformation could happen, which resulted in larger slip *s* between steel and concrete at failure. The load transfer path was extended outward at an angle. A larger region of the surrounding concrete contributed to the reactions *R*_1_ and *R*_2_. Therefore, the shear strength and the slip ductility of the perfobond connectors could be greatly enhanced by providing a rebar in hole.

### 3.3. Load–Slip Response

**Figure 6:**
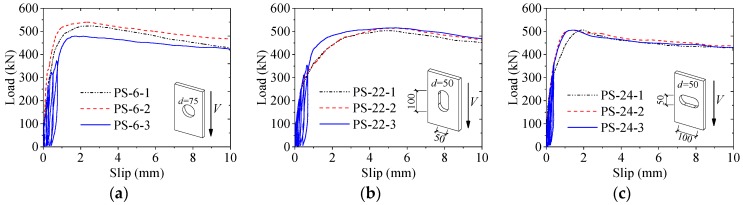
Load–slip response. (**a**) Circular hole: *d* = *d_l_* = *d_h_*; (**b**) long hole: *d* = *d_h_* < *d_l_*; (**c**) long hole: *d* = *d_l_* < *d_h_*.

As shown in Figure 6, the typical load–slip curves of the perfobond connectors consisted of a linear branch with very small slips, followed by a nonlinear branch with a peak point, and ended with a slowly descending branch. The shear strength (*V_u_*_,*i*_ and *V_u_*_,*avg*_), the shear stiffness (*k_s_*_,*i*_ and *k_s_*_,*avg*_), and the peak slip (*s_p_*_,*i*_ and *s_p_*_,*avg*_) of the perfobond connectors were derived from the tested curves and are summarized in Table 2.

### 3.4. Stiffness Variations

**Figure 7:**
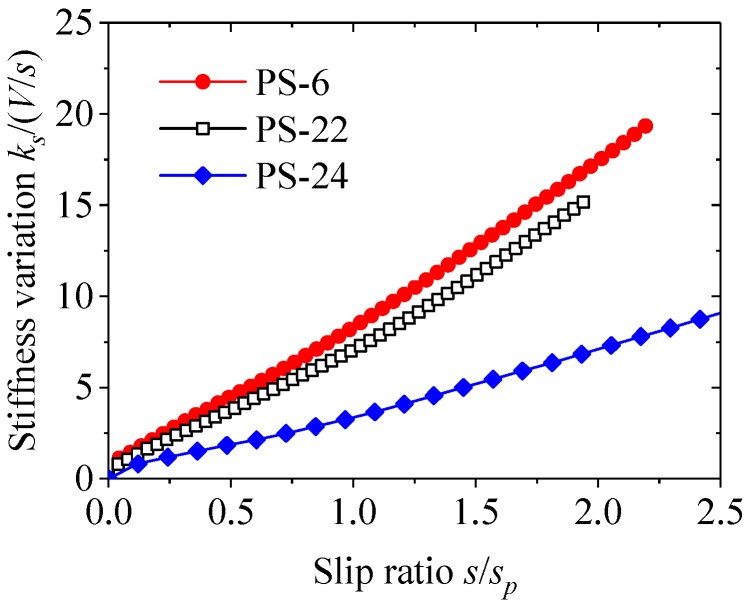
Stiffness variations.

The secant slope *V*/*s* of the load–slip curves reflected the continuous stiffness variation of the perfobond connector. In comparison, the shear stiffness *k_s_* represented the ability to resist slip deformation at the initial stage. Figure 7 shows the typical relationship between the ratio of the shear stiffness *k_s_* to the secant slope *V*/*s* and the ratio of the slip *s* to the peak slip *s_p_*. The hole shapes of the perfobond connectors in groups PS-6, PS-22, and PS-24 were circular hole (*d* = *d_l_* = *d_h_* = 75 mm), long hole (*d* = *d_h_* = 50 mm, *d_l_* = 100 mm), and wide hole (*d* = *d_l_* = 50 mm, *d_h_* = 100 mm), respectively. The hole areas of these specimens were approximate to each other regardless of the hole shape. As shown in Figure 6, the stiffness ratio *k_s_*/(*V*/*s*) increased with the increase of the slip ratio *s*/*s_p_*, indicating that the stiffness of the load–slip curve reduced continuously as the load was applied.

### 3.5. Stage Identifications

**Figure 8:**
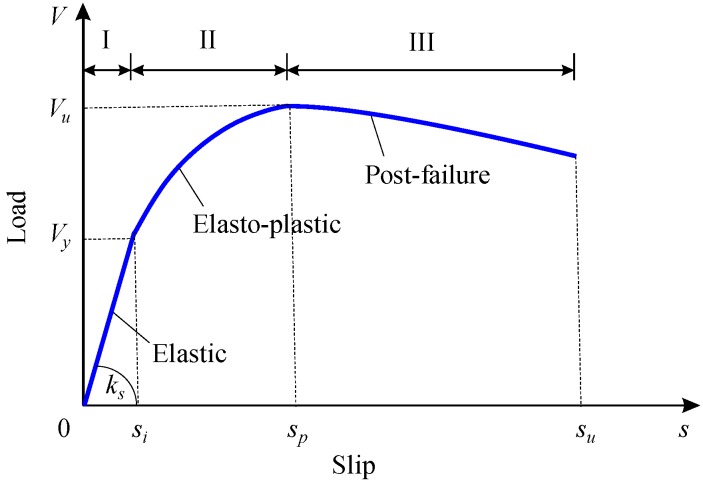
Stage identifications.

Based on the push-out test results, three stages could be identified from the typical load–slip curves of the perfobond connectors, as shown in Figure 8.

(1) Elastic stage

At the beginning of loading, the load–slip response of the perfobond connectors followed a linear elastic relationship until the value of the initial slip *s_i_* was reached. Based on the push-out test results, the initial slip *s_i_* of the perfobond connectors could be approximately quantified as 0.2 mm. The corresponding load was the yield strength *V_y_*, which was approximately 40% to 50% of the shear strength *V_u_* of the perfobond connectors. The shear stiffness *k_s_* of the perfobond connectors was defined as the secant slope *V_y_*/*s_i_* of the initial yield at the elastic stage. The upward trend of the elastic stage was controlled by the shear stiffness *k_s_*. Greater shear stiffness indicated faster growth in the shear load with the increase of slip.

(2) Elasto-plastic stage

In the following branch of load–slip curves, the shear load continued to increase until reaching the peak point. The secant slop *V*/*s* decreased with the increase of slip *s*. The nonlinear curve indicated accumulated plastic deformations in the concrete dowel and the rebar in hole. The maximum load at this stage was denoted as the shear strength *V_u_*, and the corresponding slip was denoted as the peak slip *s_p_*. The peak point of the load–slip curves was controlled by the shear strength *V_u_* and the peak slip *s_p_*.

(3) Postfailure stage

Beyond the peak point, the shear load *V* of the perfobond connectors reduced as the slip *s* continued to increase. For perfobond connectors without rebar in hole, the load–slip curves had a short descending branch, and the ultimate slip *s_u_* could be assumed as equal to the peak slip *s_p_*. When a rebar was provided in the hole, the shear load *V* reduced slowly with the increase of the slip *s*, indicating better ductility of the perfobond connectors. As the slip *s* reached 2.5 times the peak slip *s_p_*, the shear load *V* reduced by about 10% of the shear strength *V_u_*, which was consistent with the suggestion given by JSCE [23]. As a result, the ultimate slip *s_u_* was assumed to be 2.5*s_p_* for the perfobond connectors with rebar in hole.

### 3.6. Curve Characteristics

According to the stage identifications of the perfobond connectors under push-out tests, the characteristics of the load–slip curves could be expressed by the following mathematical descriptions.

(1) When $0\leq s\leq s_{u}$, $0\leq V\leq V_{u}$.

The values of the load *V* and the slip *s* were both non-negative. There existed a maximum value in each load–slip curve. The perfobond connector was subjected to uniaxial loading. When the load *V* reached the shear strength *V_u_*, the perfobond connector failed in shear.

(2) When s=0, V=0.

The origin of the load–slip curves was the zero point. The perfobond connector had no residual slip deformation and sustained no shear load before the loading began.

(3) When $0\leq s\leq s_{p}$, dV/ds≥0, and $d^{2}V/ds^{2}<0$.

Before reaching the peak point, the load *V* increased faster at first and then more slowly with the increase of the slip *s*. The branch of the load–slip curve at the elastic and elasto-plastic stages was a convex increasing curve.

(4) When $s=s_{p}$, dV/ds=0, and $V=V_{u}$.

The load–slip curve had a single peak point (*s_p_*, *V_u_*). As the slip reached the peak slip *s_p_*, the load *V* reached the maximum load *V_u_*, which was taken as the shear strength of the perfobond connector.

(5) When $s_{p}<s\leq s_{u}$, dV/ds<0.

At the postfailure stage, the load *V* decreased with the increase of the slip *s*. And when the slip *s* was greater than the ultimate slip *s_u_*, the load *V* decreased obviously, indicating that the perfobond connector was not suitable for carrying the load anymore.

## 4. Parametric Study

In steel and concrete composite structures, the shear connection behaviors are mainly characterized by the load–slip relationship of the shear connectors. The load–slip relationship could be expressed by different forms, such as load–slip curves, stiffness variations, and normalized load–slip curves. Based on the push-out test results, several parameters that affect the load–slip relationship of perfobond connectors are discussed as follows.

### 4.1. Influence of Hole Geometry

**Figure 9:**
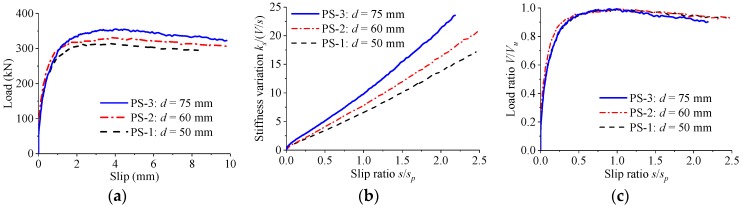
Effect of hole diameter. (**a**) Load–slip curves; (**b**) stiffness variations; (**c**) normalized curves.

Figure 9 shows the load–slip relationship of the perfobond connectors with the parameter being the hole diameter. When the hole diameter was increased from 50 mm to 60 mm and from 50 mm to 75 mm, the shear strength increased by 5% and 13%, and the peak slip increased by 13% and 32%, respectively. Regardless of the change in the hole diameter, the stiffness of the load–slip curves reduced slowly as the slip increased. Similar patterns were found for the normalized load–slip curves with different hole diameters.

**Figure 10:**
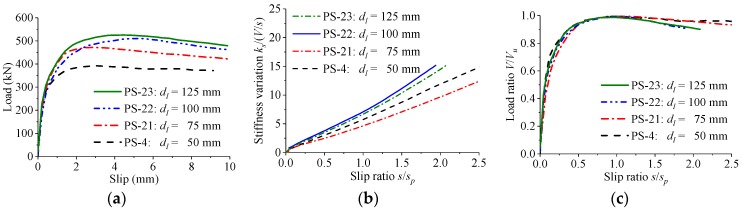
Effect of hole length. (**a**) Load–slip curves; (**b**) stiffness variations; (**c**) normalized curves.

The effect of the hole length on the load–slip relationship of the perfobond connectors is shown in Figure 10. When the hole length was increased from 50 mm to 125 mm in increments of 25 mm, the shear strength increased by 20%, 30%, and 35%, and the peak slip varied by −8%, 67%, and 56%, respectively. The stiffness of the load–slip curves reduced slowly with the slip between steel and concrete. The normalized load–slip curves with different hole lengths were almost the same.

**Figure 11:**
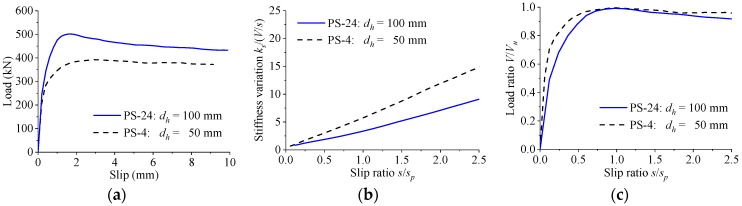
Effect of hole height. (**a**) Load–slip curves; (**b**) stiffness variations; (**c**) normalized curves.

Figure 11 shows the load–slip relationship of the perfobond connectors with the parameters being the hole height. When the hole height was increased from 50 mm to 100 mm, the shear strength increased by 28%, while the peak slip decreased by 45%. The stiffness of the load–slip curves reduced slowly with the slip despite the changes in the hole height. The shapes of the normalized curves were approximate to each other for perfobond connectors with different hole heights.

### 4.2. Influence of Concrete Strength

**Figure 12:**
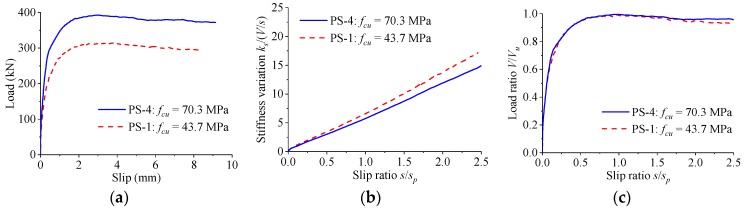
Effect of concrete strength. (**a**) Load–slip curves; (**b**) stiffness variations; (**c**) normalized curves.

As indicated in Figure 12, the concrete strength had great effect on the load–slip relationship of perfobond connectors. The increase of concrete strength from 43.7 MPa to 70.3 MPa led to an increase in the shear strength by 25% and a reduction in the peak slip by 10%. The stiffness of the load–slip curves reduced slowly with the increase of the slip. The normalized load–slip curves were almost the same, regardless of the difference in the concrete strength.

### 4.3. Influence of Rebar Configuration

**Figure 13:**
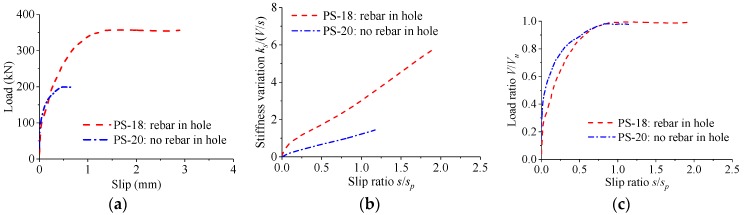
Effect of rebar’s existence in hole. (**a**) Load–slip curves; (**b**) stiffness variations; (**c**) normalized curves.

Figure 13 shows the load–slip relationship of perfobond connectors with and without rebar in hole. Compared with the specimens without rebar in hole, the shear strength and the peak slip of specimens with rebar in hole increased by 78% and 180%, respectively. As the slip increased, the stiffness of the load–slip curves reduced slowly. Similar patterns of the normalized load–slip curves were observed for perfobond connectors with and without rebar in hole.

**Figure 14:**
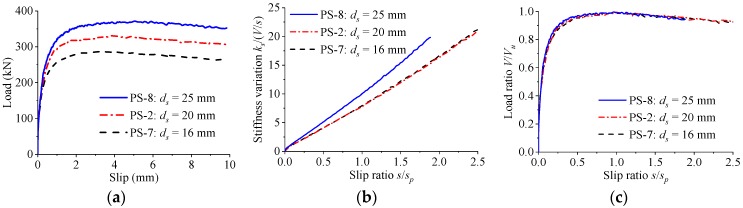
Effect of rebar diameter. (**a**) Load–slip curves; (**b**) stiffness variations; (**c**) normalized curves.

The load–slip relationships for specimens with various rebar diameters are shown in Figure 14. When the diameter of rebar in hole was changed from 16 mm to 20 mm and from 16 mm to 25 mm, the shear strength increased by 15% and 29%, while the peak slip increased by 9% and 48%, respectively. The stiffness of the load–slip curves reduced slowly with the increase of the slip. When the rebar of different diameters was provided in hole, the normalized load–slip curves almost coincided with each other.

**Figure 15:**
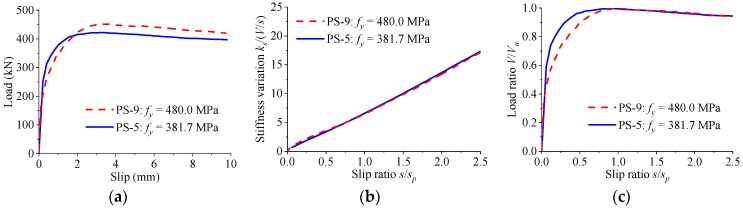
Effect of rebar strength. (**a**) Load–slip curves; (**b**) stiffness variations; (**c**) normalized curves.

Figure 15 presents the load–slip relationship for specimens with different strengths of rebar in hole. As the yield strength of rebar was increased from 381.7 MPa to 480.0 MPa, the shear strength increased by 7% and the peak slip decreased by 2%. The stiffness of the load–slip curves reduced slowly with the increase of the slip. Despite the differences in strength of rebar in hole, similar patterns of normalized curves were observed for the perfobond connectors.

### 4.4. Influence of Rib Dimension

**Figure 16:**
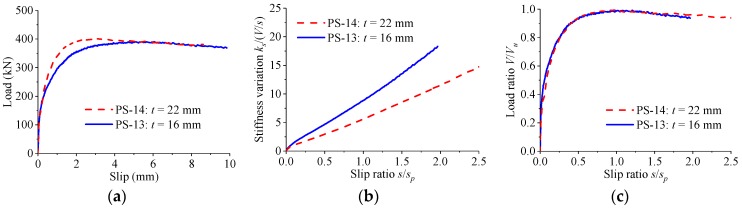
Effect of rib thickness. (**a**) Load–slip curves; (**b**) stiffness variations; (**c**) normalized curves.

The effect of the thickness of the perfobond rib on the load–slip relationship is presented in Figure 16. When the thickness of the perfobond rib was increased from 16 mm to 22 mm, the shear strength increased by less than 3%, while the peak slip decreased by 37%. Whether a thin or thick perfobond rib was used, the stiffness of the load–slip curves reduced slowly as the slip increased. Little differences were observed in the shape of normalized load–slip curves, regardless of the variations in the thickness of the perfobond rib.

**Figure 17:**
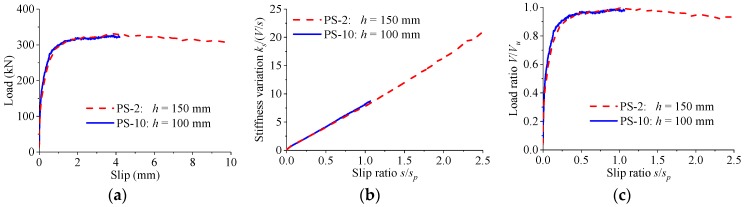
Effect of rib height. (**a**) Load–slip curves; (**b**) stiffness variations; (**c**) normalized curves.

Figure 17 shows the load–slip relationship for specimens which were identical except that the heights of the perfobond ribs were different. When the perfobond rib height was increased from 100 mm to 150 mm, the variations in the shear strength and the peak slip were both less than 2%. Despite the height of the perfobond ribs, the stiffness of the load–slip curves reduced slowly with the increase of the slip. The shapes of the normalized load–slip curves were almost the same for perfobond connectors with different rib heights.

**Figure 18:**
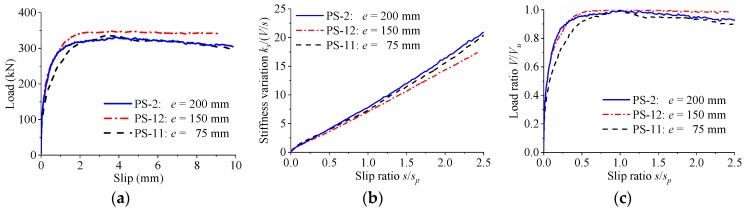
Effect of rib distance. (**a**) Load–slip curves; (**b**) stiffness variations; (**c**) normalized curves.

As revealed in Figure 18, the distance of the perfobond ribs had negligible effect on the load–slip relationship of the perfobond connectors. When the rib distance was changed among 75 mm, 150 mm, and 200 mm, the differences of the shear strength and the peak slip were less than 4%. Regardless of the differences in the rib distance, the stiffness of the load–slip curves reduced slowly as the slip increased. The pattern of the normalized load–slip curves kept almost consistent for perfobond connectors with various rib distances.

### 4.5. Influence of Slab Thickness

**Figure 19:**
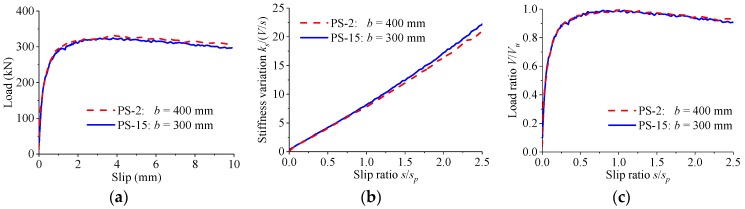
Effect of slab thickness. (**a**) Load–slip curves; (**b**) stiffness variations; (**c**) normalized curves.

Figure 19 presents the load–slip relationship for specimens which were identical except that the thicknesses of the concrete slabs were different. When the concrete slab thickness was increased from 300 mm to 400 mm, the variations in the shear strength and the peak slip of the perfobond connectors were less than 2%. As the slip increased, the stiffness of the load–slip curves reduced slowly for specimens. The normalized load–slip curves were almost the same despite the variations in the thickness of the concrete slab.

## 5. Analytical Model

### 5.1. Previous Expressions

Based on push-out tests of perfobond shear connectors which eliminated the chemical bond and the concrete-end bearing stress, JSCE [23] suggested two expressions, Equations (1) and (2), to derive the load–slip relationship of perfobond connectors without and with rebar in hole, respectively.

(1) $$\begin{array}{ll} V/V_{u} & \begin{array}{cc} ={\left[1-\exp\left(-\alpha_{0}\cdot s/d\right)\right]}^{\beta_{0}} & \left(0\leq s\leq s_{p}\right) \end{array}\\ & \mathrm{with}\alpha_{0}=500\left(t/d\right);\;\beta_{0}=1/3 \end{array}$$

(2) $$\begin{array}{ll} V/V_{u} & =\{\begin{array}{ll} {\left[1-\exp\left(-\alpha \cdot s/d_{s}\right)\right]}^{\beta } & \left(0\leq s\leq s_{p}\right)\\ {\left[1-\exp\left(-\alpha \cdot s_{p}/d_{s}\right)\right]}^{\beta }+\left(2/15\right)\left(1-s/s_{p}\right) & \left(s_{p}<s\leq 2.5s_{p}\right) \end{array}\\ & \mathrm{with}\alpha =50\left(t/d\right);\;\beta =1/3 \end{array}$$

where *α*_0_, *β*_0_, *α*, and *β* are fitting coefficients affecting the shape of load–slip curves.

Derived from the well-known formulation of the bond–slip relationship of reinforcement in concrete, FIB [24] recommended a general expression, Equation (3), to fit the tested load–slip curves of shear connectors used in steel and concrete composite structures.

(3) $$\begin{array}{cc} V/V_{u}={\left(s/s_{p}\right)}^{\gamma } & \left(0\leq s\leq s_{p}\right) \end{array}$$

where *γ* is a fitting coefficient which should be obtained from regression analysis of test results. Based on the push-out test results of perfobond connectors in this study, the value of *γ* was determined as 0.2.

### 5.2. Proposed Expression

According to the stiffness variations of load–slip curves discussed in Section 3 and Section 4, the stiffness ratio *k_s_*/(*V*/*s*) increased with the increase of the slip ratio *s*/*s_p_*. The relationship between the stiffness ratio *k_s_*/(*V*/*s*) and the slip ratio *s*/*s_p_* was a parabola-like curve. As a result, a parabolic equation, Equation (4), could be used to express the change of *k_s_*/(*V*/*s*) over *s*/*s_p_*.

(4) $$k_{s}/\left(V/s\right)=D_{1}+D_{2}\left(s/s_{p}\right)+D_{3}{\left(s/s_{p}\right)}^{2}$$

where *D*_1_, *D*_2_, and *D*_3_ are dimensionless parameters which could be derived from theoretical analysis and experimental results.

The load–slip relationship of perfobond connectors could be obtained from Equation (4) and expressed as follows.

(5) $$V=k_{s}\frac{s}{D_{1}+D_{2}\left(s/s_{p}\right)+D_{3}{\left(s/s_{p}\right)}^{2}}$$

From the first derivative of Equation (5), the tangent slope of the load–slip curves could be derived.

(6) $$\frac{dV}{ds}=k_{s}\frac{D_{1}-D_{3}{\left(s/s_{p}\right)}^{2}}{{\left[D_{1}+D_{2}\left(s/s_{p}\right)+D_{3}{\left(s/s_{p}\right)}^{2}\right]}^{2}}$$

In accordance with the curve characteristics revealed in the test results, the following conditions should be satisfied for the load–slip relationship.

(7) $${V|}_{s=s_{p}}=V_{u}$$

(8) $${\frac{dV}{ds}|}_{s=s_{p}}=0$$

The substitutions of Equations (5) and (6) into the Equations (7) and (8) yielded the following conditions for the unknown parameters *D*_1_, *D*_2_, and *D*_3_.

(9) $$D_{2}=k_{s}s_{p}/V_{u}-2D_{1}$$

(10) $$D_{3}=D_{1}$$

Then, Equations (9) and (10) were substituted into Equation (5) and the load–slip relationship could be expressed by a simpler equation with only one unknown parameter *D*_1_.

(11) $$V=k_{s}\frac{s}{D_{1}{\left(1-s/s_{p}\right)}^{2}+k_{s}s/V_{u}}$$

Based on Equation (11), nonlinear regression analysis was carried out on the test results, and the best fit of the unknown parameter *D*_1_ was determined as 0.5. Thus, the load–slip relationship of perfobond connectors could be expressed by the following equation.

(12) $$V=k_{s}\frac{s}{0.5{\left(1-s/s_{p}\right)}^{2}+k_{s}s/V_{u}}$$

According to the stage identifications of the load–slip curves, the initial slip *s_i_* of perfobond connectors could be assumed as 0.2 mm. The corresponding yield strength *V_y_* was approximate to 0.5*V_u_*. As a result, the shear stiffness *k_s_* could be estimated by Equation (13).

(13) $$k_{s}=0.5V_{u}/s_{i}$$

Substituting Equation (13) into Equation (12), the expression of the load–slip relationship could be further simplified as follows.

(14) $$\begin{array}{cc} V=\frac{V_{u}}{1+\left(s_{i}/s\right){\left(1-s/s_{p}\right)}^{2}} & \left(0\leq s\leq s_{u}\right) \end{array}$$

where *s_i_* is the initial slip (mm), for push-out tests of the perfobond connectors, *s_i_* is assumed to be 0.2 mm; *s_p_* is the peak slip (mm); and *s_u_* is the ultimate slip (mm), without rebar in hole, *s_u_* = *s_p_*, while with rebar in hole, *s_u_* = 2.5*s_p_*.

### 5.3. Comparison and Validation

**Figure 20:**
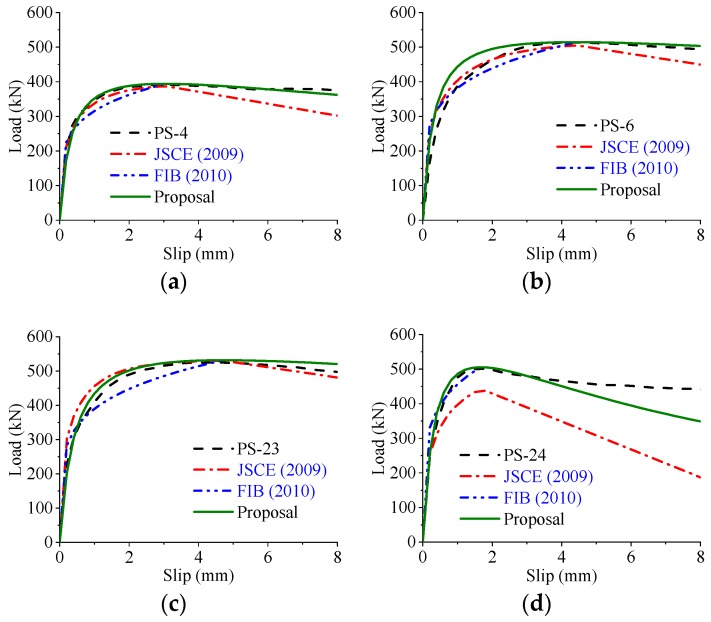
Predicted and tested curves varying hole geometry. (**a**) Smaller hole: *d* = *d_l_* = *d_h_* = 50 mm; (**b**) larger hole diameter: *d* = 75 mm; (**c**) larger hole length: *d_l_* = 125 mm; (**d**) larger hole height: *d_h_* = 100 mm.

**Figure 21:**
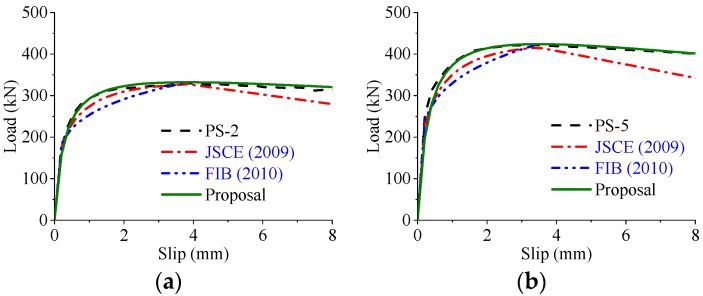
Predicted and tested curves varying concrete strength. (**a**) Lower concrete strength: *f_cu_* = 43.3 MPa; (**b**) higher concrete strength: *f_cu_* = 70.3 MPa.

**Figure 22:**
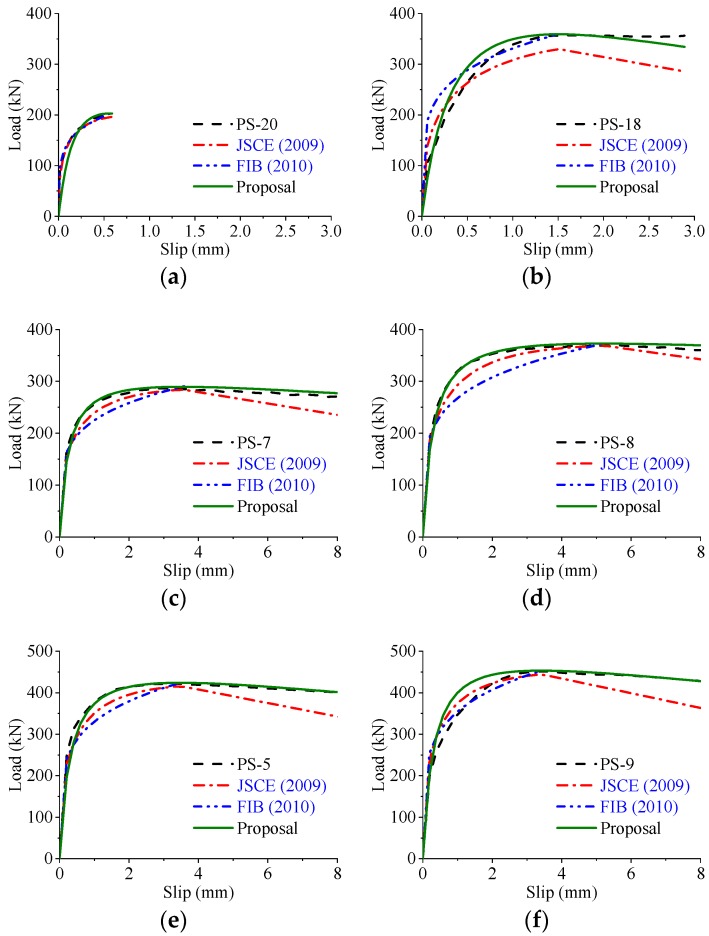
Predicted and tested curves varying rebar configuration. (**a**) Without rebar in hole; (**b**) with rebar in hole; (**c**) smaller rebar diameter: *d_s_* = 16 mm; (**d**) larger rebar diameter: *d_s_* = 25 mm; (**e**) lower rebar strength: *f_y_* = 391.7 MPa; (**f**) higher rebar strength: *f_y_* = 480.0 MPa.

Based on the parametric study, the load–slip curves of the perfobond connectors were significantly influenced by the hole geometry, the concrete strength, and the configuration of the rebar in hole. To compare the different expressions in reproducing the load–slip curves of the perfobond shear connectors, the same actual shear strength obtained from test results were used in the calculations. As shown in Figure 20, Figure 21 and Figure 22 the expressions suggested by JSCE [23] and FIB [24] agreed reasonably with the tested curves in the elastic stage. However, the shear strength was underestimated in the elasto-plastic stage. The expression of FIB [24] provided no curves for the postfailure stage, while the predicted curves of JSCE [23] reduced sharper than the tested curves in the descending branch. In comparison, the proposed expression, Equation (14), provided a better prediction of load–slip relationships with the tested curves, regardless of the variations in these critical parameters.

As shown in Table 3, different expressions for the load–slip relationship of the perfobond connectors were compared in terms of stage identifications, curve characteristics, and stiffness variations. Three stages of the load–slip relationship were included in the expressions suggested by JSCE [23], FIB [24], and the proposal, except that the postfailure stage was neglected by the expression of FIB [24]. The curve characteristics (1), (2), and (3) were all met by these three expressions. The expressions suggested by JSCE [23] and FIB [24] did not satisfy the characteristics (4), indicating that the actual shear strength was unable to be reached unless the slip increased to infinitely great. Without the postfailure stage, the curve characteristics (5) were not considered by the expression of FIB [24]. Overall, the expressions suggested by JSCE [23], FIB [24], and the proposal all agreed reasonably well with the test results. However, the proposal in this study had a better fit in terms of the individual similarity, because the stiffness variations of each load–slip curve were considered in this expression.

## 6. Conclusions

Seventy-two push-out tests of perfobond connectors were carried out. Based on the test results, parametric study, and analytical analysis, the following conclusions can be drawn.

(1) The failure modes of perfobond connectors involve cracking in the concrete slab, yielding of the rebar in hole, and shearing in the concrete dowel. No obvious deformation occurs in the structural steel and the hole edge of the perfobond rib.
(2) The shear mechanism indicates that the shear strength and the slip ductility of perfobond connectors can be greatly enhanced by providing a rebar in hole. The main reason is that the rebar in hole provides resistance to the bending moment and extends the load transfer path to the surrounding concrete.
(3) The load–slip relationship of perfobond connectors consists of a linear branch with very small slips, followed by a nonlinear branch with a peak point, and ends with a slowly descending branch. Three stages can be accordingly identified as elastic stage, elasto-plastic stage, and postfailure stage.
(4) In typical push-out tests, the load–slip curves have five characteristics which can be expressed by mathematical descriptions. These curve characteristics can be used to establish the theoretical expression for the load–slip relationship of perfobond connectors.
(5) According to the results of parametric study, the load–slip relationship of perfobond connectors is significantly influenced by the hole geometry, the concrete strength, and the configuration of the rebar in hole. The dimensions of the perfobond rib and the size of the concrete slab have negligible effect on the load–slip relationship of perfobond connectors.
(6) Based on theoretical analysis and experimental results, an analytical model is proposed to express the load–slip relationship of perfobond connectors. Compared with existing expressions, the proposed expression has more explicit physical meanings and fits better with the experimental results. Therefore, the proposed expression can be used to calculate the nonlinear behavior of perfobond connectors in composite structures.

The overall investigation may provide reference for the design and construction of shear connectors in steel and concrete composite structures.

## Figures and Tables

**Table 1:** Push-out test specimens.

Group	*d* (mm)	*d_l_* (mm)	*d_h_* (mm)	*d_s_* (mm)	*f_cu_* (MPa)	*f_y_* (MPa)	*t* (mm)	*h* (mm)	*e* (mm)	*a* (mm)	*b* (mm)
PS-1	50	50	50	20	43.3	373.6	20	150	200	460	400
PS-2	60	60	60	20	43.3	373.6	20	150	200	460	400
PS-3	75	75	75	20	43.3	373.6	20	150	200	460	400
PS-4	50	50	50	20	70.3	381.7	20	150	200	460	400
PS-5	60	60	60	20	70.3	381.7	20	150	200	460	400
PS-6	75	75	75	20	70.3	381.7	20	150	200	460	400
PS-7	60	60	60	16	43.3	373.6	20	150	200	460	400
PS-8	60	60	60	25	43.3	373.6	20	150	200	460	400
PS-9	60	60	60	20	70.3	480.0	20	150	200	460	400
PS-10	60	60	60	20	43.3	373.6	20	100	200	460	400
PS-11	60	60	60	20	43.3	373.6	20	150	75	460	400
PS-12	60	60	60	20	43.3	373.6	20	150	150	460	400
PS-13	65	65	65	20	43.3	373.6	16	210	200	460	400
PS-14	65	65	65	20	43.3	373.6	22	210	200	460	400
PS-15	60	60	60	20	43.3	373.6	20	150	200	460	300
PS-16	75	75	75	20	63.4	335.0	20	150	150	400	500
PS-17	50	50	50	20	54.6	335.0	20	100	150	400	200
PS-18	50	50	50	20	54.6	335.0	20	100	150	400	200
PS-19	50	50	50	20	54.6	335.0	20	100	150	400	200
PS-20	50	50	50	—	54.6	335.0	20	100	150	400	200
PS-21	50	75	50	20	70.3	381.7	20	150	200	460	400
PS-22	50	100	50	20	70.3	381.7	20	150	200	460	400
PS-23	50	125	50	20	70.3	381.7	20	150	200	460	400
PS-24	50	50	100	20	70.3	381.7	20	150	200	460	400

**Table 2:** Push-out test results.

Group	Shear Strength *Vu* (kN)				Shear Stiffness *ks* (kN·mm^−1^)				Peak Slip *sp* (mm)			
	*V_u,_*_1_	*V_u,_*_2_	*V_u,_*_3_	*V_u,avg_*	*k_s,_*_1_	*k_s,_*_2_	*k_s,_*_3_	*k_s,avg_*	*s_p,_*_1_	*s_p,_*_2_	*s_p,_*_3_	*s_p,avg_*
PS-1	328.0	306.9	314.3	316.4	629.5	571.0	624.7	608.4	3.22	3.28	3.68	3.39
PS-2	335.3	329.0	332.0	332.1	690.0	607.5	717.9	671.8	3.53	4.14	3.85	3.84
PS-3	386.2	333.8	353.4	357.8	797.1	786.1	744.1	775.8	3.86	4.43	5.18	4.49
PS-4	386.9	430.5	364.9	394.1	720.1	732.2	771.4	741.2	3.13	2.95	3.03	3.04
PS-5	420.0	438.5	413.5	424.0	789.6	805.2	795.8	796.9	3.86	3.25	3.33	3.48
PS-6	523.6	540.1	479.5	514.4	988.4	986.7	924.7	966.6	4.17	4.68	4.41	4.42
PS-7	284.9	284.4	299.1	289.5	633.4	678.5	621.0	644.3	3.37	3.94	3.28	3.53
PS-8	397.0	346.1	375.4	372.8	732.4	715.4	681.1	709.7	5.55	5.18	4.94	5.22
PS-9	440.0	466.0	454.0	453.3	843.7	856.0	865.9	855.2	3.34	3.22	3.70	3.42
PS-10	350.5	317.1	321.1	329.6	688.4	698.4	626.7	671.1	3.75	3.88	4.17	3.93
PS-11	335.3	333.8	347.5	338.9	711.3	641.3	638.2	663.6	3.61	3.48	4.03	3.71
PS-12	362.7	358.3	318.7	346.6	621.7	602.1	713.0	645.6	4.07	3.50	3.82	3.80
PS-13	392.6	394.5	394.0	393.7	648.6	712.8	697.9	686.5	4.55	5.03	5.45	5.01
PS-14	374.0	415.6	422.4	404.0	712.5	723.3	687.1	707.6	3.27	3.13	3.08	3.16
PS-15	323.6	334.6	322.1	326.8	683.4	618.5	711.4	671.1	3.81	4.22	3.74	3.92
PS-16	494.1	474.9	515.0	494.7	1114.9	963.3	—	1039.1	2.79	2.48	—	2.64
PS-17	340.7	320.0	434.0	364.9	738.9	728.2	783.3	750.1	2.18	0.36	1.74	1.43
PS-18	362.8	375.9	339.2	359.3	722.2	751.8	678.8	717.6	1.62	1.32	1.56	1.50
PS-19	363.0	351.0	362.2	358.7	700.9	742.7	753.6	732.4	1.89	1.91	1.78	1.86
PS-20	204.7	167.2	237.5	203.1	417.0	405.8	454.3	425.7	0.65	0.57	0.48	0.57
PS-21	501.0	442.5	477.0	473.5	856.9	711.4	790.2	786.2	2.72	3.06	2.59	2.79
PS-22	503.0	514.6	515.6	511.1	668.7	679.3	782.7	710.2	4.83	5.41	5.02	5.09
PS-23	477.0	556.6	562.1	531.9	702.0	733.3	788.7	741.3	4.75	4.09	5.36	4.73
PS-24	506.5	505.5	505.0	505.7	991.6	1034.0	1003.3	1009.6	1.99	1.41	1.60	1.67

**Table 3:** Comparison of expressions for load–slip relationship.

Consideration for Key Features		JSCE [23]	FIB [24]	Proposal
Stage identifications	(1) Elastic stage	√	√	√
	(2) Elasto-plastic stage	√	√	√
	(3) Postfailure stage	√	×	√
Curve characteristics	(1) When $0\leq s\leq s_{u}$, $0\leq V\leq V_{u}$.	√	√	√
	(2) When s=0, V=0.	√	√	√
	(3) When $0\leq s\leq s_{p}$, $\frac{dV}{ds}\geq 0$ and $\frac{d^{2}V}{ds^{2}}<0$.	√	√	√
	(4) When $s=s_{p}$, $\frac{dV}{ds}=0$, and $V=V_{u}$.	×	×	√
	(5) When $s_{p}<s\leq s_{u}$, $\frac{dV}{ds}<0$.	√	×	√
Stiffness variations	(1) Overall similarity	√	√	√
	(2) Individual similarity	×	×	√
